# Urotropine: A Urinary Antiseptic

**Published:** 1901-01-26

**Authors:** 


### UROTROPINE: A URINARY ANTISEPTIC.

MUCH as we may doubt the possibility of obtaining by the use of drugs any useful degree of internal antisepsis, so far as concerns the intestinal canal, the case is somewhat different in regard to the urinary passages. The old remedies, benzoic acid and benzoate of ammonia, were not things on which much reliance could be placed, yet in certain cases one could hardly doubt that they had an effect in at least exercising some inhibitory influence over bacterial growth. Again the very striking effect of copaiba in certain cases of gonorrhœa can hardly be explained except on the hypothesis that the drug so far as it is excreted by the urine has an influence in checking the growth of the gonococcus in and on the mucous membrane. Urotropine, however, is the drug which seems to give the most striking evidence of its power over certain microbic infections of the urinary passages. This drug, otherwise called hexamethylene-tetramine ($C_6H_{12}N_4$), is a white crystalline body produced by the action of ammonia on formaldehyde. It was introduced in 1894 by Professor Nicolaier as a diuretic and a solvent of uric acid, and he also claimed that it would prevent the development of bacteria in the urine. Lately it has been shown by several observers to have a marked effect in putting a stop to the development of the typhoid bacilli in the urine, which in some patients affected with enteric fever gives rise to a veritable bacilluria, the urine being rendered semi-opaque by the presence of these organisms; and Dr. Horton Smith has especially laid stress upon the power of urotropine in controlling this condition. Mr. P. J. Cammidge[1] has lately investigated the whole subject, and he has more especially endeavoured to find out what is the active bactericidal agent by which the result is obtained, one of the questions to which he more particularly directed his inquiries being how far the bactericidal effect of urotropine might be due to its becoming decomposed, and giving off among other things formaldehyde. The results of his researches go to show that, although the exact chemical nature of the antiseptic body occurring in the urine under the use of urotropine has not been definitely settled, it seems clear that it is not free formaldehyde. On the other hand it would seem that it is not the urotropine itself which is the most important factor in producing this kolyseptic action. It is probable that acid urines produce in the kidney a partial decomposition of the urotropine by which some body is liberated or some fresh compound formed which has a marked inhibitory power over the growth of bacteria. An important point then in securing the full effect of the drug in bacterial infections of the urine is to ensure that the urine should be acid in reaction as it leaves the kidney. Take the matter altogether, it appears that urotropine is a much better urinary antiseptic than those usually employed, especially when acidity of the urine can be ensured. Its administration has been highly recommended by Caspar as a means of procuring an aseptic condition of the genito-urinary tract in operations on those regions. Bacteruria of various kinds and the nocturnal enuresis of children, which is said to be frequently caused by infection of the urine with bacillus coli, would probably be benefited by treatment with urotropine, and in typhoid fever it may not only be employed for the treatment of typhoid bacilluria, for which it is almost a specific, but its routine employment from the third or fourth week onward is to be recommended as a preventive of that prolonged infection of the urine which, when it continues after discharge from hospital, constitutes a real danger to the public.

[1] Lancet, Jan. 19.

